# Deep Learning for Emergency Department Sustainability: Interpretable Prediction of Revisit

**DOI:** 10.3390/healthcare14040464

**Published:** 2026-02-12

**Authors:** Wang-Chuan Juang, Zheng-Xun Cai, Chia-Mei Chen, Zhi-Hong You

**Affiliations:** 1 Quality Management Center, Kaohsiung Veterans General Hospital, Kaohsiung 813114, Taiwan; 2 Department of Business Management, National Sun Yat-sen University, Kaohsiung 804201, Taiwan; 3 Department of Health-Business Administration, Fooyin University, Kaohsiung 831801, Taiwan; 4 Department of Information Management, National Sun Yat-sen University, Kaohsiung 804201, Taiwan

**Keywords:** deep learning, model interpretability, unscheduled return visit, sustainability of emergency operations, reattendance, emergency department

## Abstract

Background: Emergency department (ED) overcrowding strains clinicians and potentially compromises urgent care quality. Unscheduled return visits (URVs), also known as readmissions, contribute to this cycle, motivating tools that identify high-risk patients at discharge. Methods: This study performed a retrospective study using ED electronic health records (EHRs) from Kaohsiung Veterans General Hospital from January 2018 to December 2022 (n = 184,653). The model integrates structured variables, such as vital signs, medication and laboratory counts, and ICD-10–based comorbidity measures, with unstructured physician notes. Key physiologic measurements were transformed into binary form using clinical reference intervals, and random under-sampling addressed class imbalance. A multimodal, CNN was proposed and evaluated with an 8:2 train–test split and 10-fold Monte Carlo cross-validation. Results: The proposed model achieved a sensitivity of 0.717 (CI: [0.695, 0.738]), accuracy of 0.846 (CI: [0.842, 0.850]), and AUROC of 0.853. Binary transformation improved recall and AUROC relative to the original numeric representations. SHAP analysis showed that unstructured features dominated prediction, while structured variables added complementary value. In a small-scale pilot evaluation using the SHAP-enabled interface, participating physicians reported the system helped surface high-risk cohorts and reduced cognitive workload by consolidating relevant patient information for rapid cross-checking. Conclusions: An interpretable CNN-based clinical decision support system can predict ED revisit risk from multimodal EHR data and demonstrates practical usability in a real-world clinical setting, supporting targeted discharge planning and follow-up as a near-term approach to mitigate overcrowding.

## 1. Introduction

The emergency department plays an important role in modern healthcare systems and serves as the primary entry points for patients requiring urgent and sometimes life-saving medical treatments. Besides, the ED functions as not only a critical safety net for vulnerable populations but a gateway to expand hospital services. Given the centrality of the ED in ensuring timely and equitable access to medical care, it is crucial to maintain the sustainability of ED operations, which requires holding the capacity to meet the current demands without compromising the quality of services and possess the ability to adapt and remain resilient in the face of multifaceted difficulties, such as growing patient volumes, resource limitations, and evolving public health challenges.

ED overcrowding has emerged as one of the most pressing threats to this sustainability, as it significantly decreases both the quality of patient care and the operational efficiency of healthcare systems. An overcrowded ED not only strains medical resources but also significantly overloads clinical staff, leading to decision-making pressure and potential for medical errors. In such environments, individual patients often receive reduced attention and diagnostic time, which may result in suboptimal clinical assessments and delayed interventions. These factors increase the likelihood of patients returning to the ED for unresolved or worsening conditions, exacerbating the overcrowding cycle.

Overcrowded EDs have become a prevalent issue globally, with recent years witnessing a substantial increase in patient volumes and corresponding demands on healthcare resources [1]. Statistical data from Taiwan indicate that ED visits exceeded 6.1 million in 2022, representing a 13.6% increase compared to 2021 figures [2]. This surge includes patients presenting with a wide range of disease severity and comorbidities, further intensifying the demand for medical services within the ED. As the utilization of emergency care continues to rise, the issue of unexpected/unscheduled return visits (URVs) poses a growing challenge, testing the capacity, efficiency, and quality of ED operations.

In Taiwan, ED overcrowding constitutes a common phenomenon across medical institutions, attributable to patients’ frequent preference for seeking care at major medical centers rather than local clinics. This tendency is mainly influenced by the presence of the National Health Insurance (NHI) system, under which the cost of diagnostic services is relatively comparable across different levels of healthcare institutions. As a result, patients with non-emergent conditions often bypass community-based clinics and directly present to emergency departments without appropriate referrals, contributing to the unnecessary utilization of emergency services. Furthermore, the capacity of primary care providers to appropriately refer patients to specialized departments or tertiary hospitals is often hindered by limited communication channels, insufficient follow-up mechanisms, and the absence of standardized referral protocols. In such contexts, many conditions that could be effectively managed at the primary care level are inappropriately escalated to emergency care settings, compounding the burden on ED resources and worsening issues of overcrowding and care inefficiency.

Addressing the issue of ED overcrowding requires the implementation of multifaceted, long-term strategies aimed at optimizing healthcare resource distribution and enhancing patient flow management. Although systemic reforms such as improving primary care accessibility and reinforcing referral mechanisms are crucial, their implementation often requires considerable time, administrative restructuring, and policy-level adjustments. In response to the need for more immediate and practical relief, this study proposes a clinical decision support system (CDSS) to predict the likelihood of patient unplanned revisits. By incorporating predictive analytics, the proposed CDSS facilitates the early identification of high-risk patients, which enables the deployment of targeted measures, including individualized discharge planning and structured follow-up care. These interventions not only contribute to improved patient outcomes but also serve to reduce the operational burden on EDs, offering a scalable and effective approach to mitigating overcrowding while broader structural reforms are pursued.

The application of information technology to enhance healthcare delivery has been recognized for decades. To mitigate risks and minimize clinical errors, healthcare organizations frequently rely on retrospective event analysis. Health information technologies, such as electronic health records (EHRs), enable efficient access to historical patient data. With the increasing adoption of machine learning (ML) and deep learning (DL) for outcome prediction, prior research has demonstrated its applicability in assessing patient risk using large-scale data repositories. Building on this foundation, the present study developed a DL-assisted model to predict unexpected return visits in the ED, with the expectation that such an approach could enhance patient safety and reduce medical expenditures by lowering the frequency of URVs.

The remainder of this paper is organized as follows. Section 2 (Literature Review) contextualizes the challenges of predicting URVs and discusses existing predictive approaches. Section 3 (Methodology) details the data selection process and the technical architecture of the proposed CNN framework. The experimental findings, including the performance evaluation and the interpretable SHAP-based interface, are presented in Section 4 (Results). Section 5 (Discussion) interprets the results through a clinical lens and evaluates the system’s utility based on physician feedback. Section 6 (Research Limitations & Future Work) addresses the constraints of the current study and outlines directions for subsequent refinement. Finally, the Conclusions synthesizes our primary contributions and offers concluding remarks on the framework’s potential for real-world clinical implementation.

## 2. Literature Review

### 2.1. Background

An unexpected or unscheduled return visit is a situation where patients return to the same department or hospital because their clinical symptoms have not experienced adequate improvement, or in some cases, have deteriorated further. A high incidence of URV poses significant challenges to the ED sustainability [3]. Specifically, it increases the overall workload for ED clinicians, which in turn intensifies challenges associated with resource allocation and staff fatigue. Moreover, the additional patient volume resulting from URV inevitably constrains the time available for diagnostic evaluation and therapeutic decision-making on an individual basis. Such constraints may further compromise the quality of care delivery, hinder optimal clinical outcomes, and create a cyclical burden that undermines both patient safety and the operational efficiency of the ED.

In order to mitigate the occurrence of URV, Gelder et al. [4] conducted a study investigating predictors of 30-day ED revisits as well as 90-day functional decline or mortality. Their findings indicated that age, sex, polypharmacy, and cognitive impairment were independent predictors of a 30-day ED revisit. However, they also concluded that the development of an effective clinical prediction model based on statistics may not be feasible. Another similar study proposed by Duseja et al. [5] analyzed multi-state ED revisit data and reported that 8.2% of patients returned within 72 h of an index ED visit, with 32% of these revisits occurring at a different healthcare institution. Furthermore, revisit rates were shown to vary depending on both the underlying diagnosis and the state in which care was provided.

Several studies have provided comprehensive analyses of ED revisit cases in Taiwan. Wu et al. [6] reported that 5.47% of patients experienced URV within 72 h, and the majority of these revisits was attributed to worsened clinical conditions. Among the presenting symptoms, abdominal pain accounted for the largest proportion. Similarly, Lin et al. [7] analyzed multiple URV cases related to abdominal illness and concluded that older patients, particularly those prescribed multiple analgesics and subjected to simultaneous laboratory examinations, showed a higher likelihood of returning unexpectedly to the ED. Expanding on these findings, Guo et al. [8] examined URV from multiple perspectives and identified several contributing factors, including blood pressure, pulse rate, fever, triage level, gender, and primary illness. Among these variables, advanced age emerged as the most significant determinant of URV.

### 2.2. ML-Based Approaches

With the rapid advancement of ML methodologies, Lee et al. [9] conducted a review of recent studies that employed ML models as primary tools for predicting ED revisits. Their analysis revealed that logistic regression remains the most commonly applied algorithm in this context, although Extreme Gradient Boosting generally demonstrated superior predictive performance. Building on this trend, Martini et al. [10] implemented an enhanced variant of Decision Trees, namely Gradient Boosting Decision Trees, within a real-time URV prediction framework, achieving an area under the curve (AUC) of 0.88 for hospital admission prediction across pooled test datasets. In a related investigation, Bleich et al. [11] applied Random Forest models to predict URV within both 7-day and 30-day intervals. Their findings indicated moderate predictive performance, with a sensitivity and specificity of 65% and 71% for the 30-day model, and 62% and 66% for the 7-day model, respectively.

### 2.3. DL-Based Approaches

Although ML-based models can achieve acceptable levels of performance, DL offers distinct advantages by not only capturing trends and patterns within the data but also uncovering complex relationships among variables. Given that EHR data consist of both structured and unstructured formats, DL-based approaches are particularly advantageous, as they can effectively leverage information from both forms of data without requiring extensive efforts to transform unstructured data into structured representations.

Davazdahemami et al. [12] employed a Deep Neural Network, a representative DL-based algorithm, within their predictive framework to analyze both structured and unstructured EHR data. Their findings indicated that the incorporation of unstructured information, such as physicians’ notes, substantially improved model performance compared to approaches that relied solely on structured data during training. Chen et al. [13] also reached a similar conclusion. By integrating clinicians’ notes into a clinical narrative-aware pretrained language model, BlueBERT, they were able to extract critical factors from the textual data. Their study demonstrated that the BERT-based model outperformed prior patient disposition prediction models, thereby showing its potential utility in ED clinical practice. Saggu et al. [14] developed and evaluated GNN and RNN models to predict ED revisit within a 30-day period among child and youth mental health outpatients. Their experimental results demonstrated that the GNN-based model exhibited superior detection performance over RNN-based techniques.

## 3. Methodology

To mitigate the risk of URV, it is essential for emergency clinicians to exercise careful judgment when making discharge decisions. In this study, DL techniques were employed to develop a CDSS for URV prediction, with the aim of assisting clinicians by estimating the likelihood of URV for individual ED patients. Such an approach has the potential to improve patient outcomes and reduce avoidable ED readmissions.

The collected EHR encompassed both structured and unstructured data. The structured component consisted of patients’ vital signs in the ED, including blood pressure, respiratory rate, and body temperature, whereas the unstructured component primarily comprised textual information such as clinicians’ notes. Prior studies have demonstrated that integrating both structured and unstructured data can improve predictive performance, as DL models are capable of capturing latent patterns embedded within unstructured information. Building upon this evidence, the present study utilized retrospective administrative medical records of patients who visited the ED of the collaborating hospital between January 2018 and December 2022. Table 1 illustrates the demographic of the structured variables in the studied dataset.

**Figure 1:**
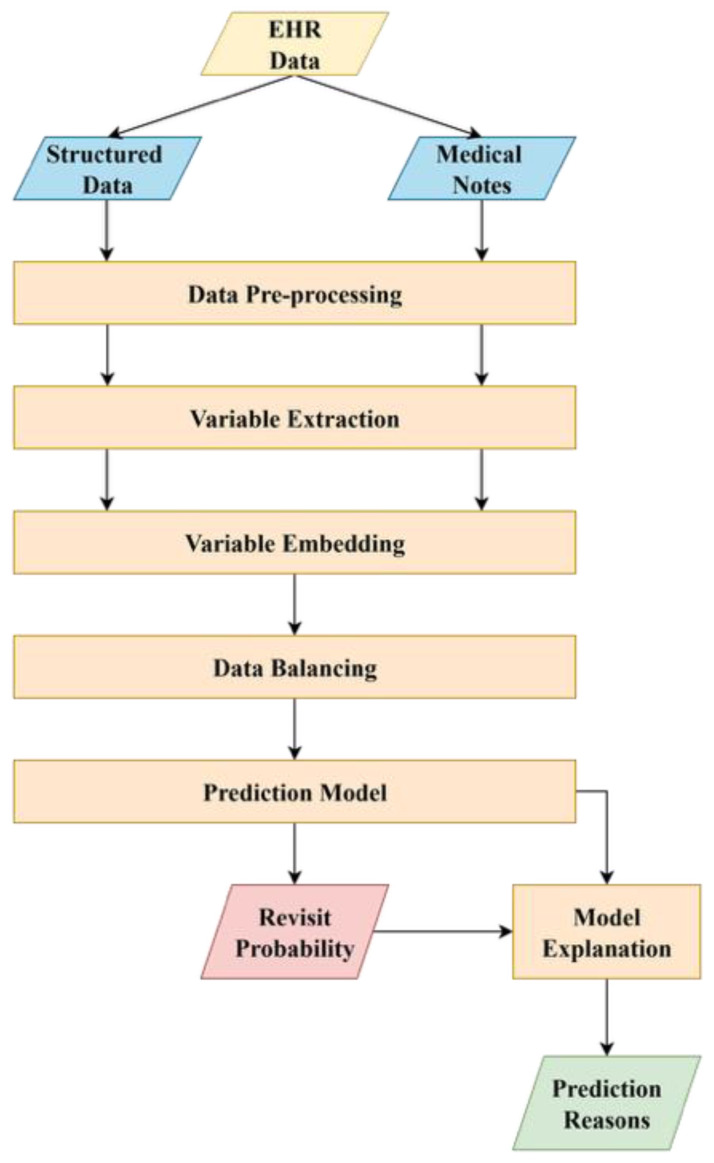
System architecture of the proposed CDSS. Rectangles represent processing modules, while parallelograms indicate data inputs and output results.

Figure 1 outlines the proposed system architecture consisting of the following modules: data pre-processing, variable extraction, variable embedding, data balancing, prediction model, and model interpretation. Each is explained in the following subsections.

### 3.1. Data Preprocessing

Data preprocessing represents a critical step to ensure the accuracy and consistency of the collected data. In this study, each medical record was examined for missing or potentially erroneous values. The preprocessing module was designed to perform comprehensive data cleansing on both structured and unstructured datasets prior to downstream analysis.

For the structured data, records containing incomplete or missing values were excluded from the study for several considerations. First, incomplete entries may introduce noise and bias, thereby degrading the predictive performance and robustness of deep learning or machine learning models. Second, commonly adopted imputation techniques, such as interpolation or value substitution, are often unsuitable in medical settings, as artificially generated values may represent clinically implausible conditions and thus convey misleading or entirely different medical meanings. Third, the exclusion of incomplete records was deemed to have a minimal impact on the overall dataset, as such records constituted only a small proportion of the collected data and their removal was therefore unlikely to compromise the representativeness of the study population.

The unstructured data in this study consisted primarily of free-text medical notes authored by emergency department physicians. These notes encompassed rich clinical information, including the patients’ medical histories, presenting complaints, symptom descriptions, and reasons for admission. Preliminary analyses indicated that converting these narrative records into a fully structured format was impractical due to substantial variability in the physicians’ documentation styles, terminology usage, and narrative organization. In order to preserve the semantic integrity and clinical nuance of the original records, the unstructured data were retained in their textual form. The only modification applied was the translation of notes originally written in Mandarin into English using the Google Translate service, which was performed to ensure linguistic consistency across the dataset and to facilitate subsequent analysis.

### 3.2. Variable Extraction

This module enriches the variable set by generating composite variables from the combination of two or more existing attributes. For instance, while body height and weight may have limited predictive value when considered independently, the Body Mass Index (BMI), calculated from both variables, provides greater clinical relevance and predictive capacity.

This study additionally computed the mean arterial pressure (MAP) using both systolic and diastolic blood pressure values and attained the Charlson Comorbidity Index (CCI) value by incorporating multiple relevant variables. The collected dataset included ICD-10 codes, which provide standardized disease classifications; however, this field typically recorded only the primary diagnosis as determined by the attending physician. In contrast, the medical notes offered more comprehensive descriptions of the patients’ comorbid conditions. Accordingly, this study extracted disease information from both the ICD-10 field and the unstructured medical notes, and subsequently calculated the CCI for each record based on the required variables.

In addition, due to the high heterogeneity of textual information related to prescribed medications and laboratory examinations, categorizing or standardizing these variables was considered impractical. To address this challenge, the proposed framework quantified such information by counting the number of distinct medication types prescribed to each patient and the number of examinations received. This transformation effectively reduces data complexity while preserving comparable predictive capacity, which can facilitate more efficient model training.

### 3.3. Variable Embedding

Variables can have different embedding schemes that may influence predictive performance. Most prior research applied standardization or normalization techniques to rescale numeric variables into a range between 0 and 1. However, such normalization may reduce clinically meaningful distinctions or even mislead the predictive model. For example, in clinical practice, body temperatures ranging from 35 °C to 37 °C are generally regarded as physiologically normal, whereas values below 35 °C or exceeding 37 °C are commonly interpreted as abnormal. When normalization is applied, these clinically defined boundaries are collapsed into continuous, rescaled values, thereby diminishing the semantic distinction between normal and abnormal physiological states. As a result, clinically salient information embedded in absolute measurement values may be lost, potentially misleading the predictive model and undermining its ability to capture medically relevant patterns.

Therefore, the proposed framework transforms patients’ vital signs into binary variables representing “normal” and “abnormal” categories, as demonstrated in Table 2. Values falling within the clinically defined normal range are embedded as 0, and as 1 otherwise. This approach preserves the clinical significance of vital signs and minimizes potential biases introduced by numeric discrepancies.

One-hot encoding (OHE) is a widely adopted approach for processing categorical variables, as demonstrated in Table 3. This technique transforms multi-valued attributes, such as symptoms, into independent binary variables, with each variable indicating whether a specific attribute is present or absent. By employing this representation, the model can effectively capture categorical information without imposing an artificial ordinal relationship among categories. Furthermore, this transformation facilitates the integration of categorical and numerical features within the same analytical framework, thereby enhancing the overall robustness of predictive modeling. In this study, one-hot encoding was applied to symptom-related attributes and other categorical variables to ensure consistency across the dataset and to preserve clinically meaningful distinctions among categories.

### 3.4. Data Balancing

A balanced, or at least near-balanced, class distribution is essential for building an effective prediction model, particularly when employing DL algorithms [15]. However, the collected medical records are real-world cases from the collaborating hospital and exhibit an inherent class imbalance, where ED readmission cases account for only a small proportion. To mitigate the negative effects of such imbalance on predictive performance, data resampling techniques are applied widely, either by augmenting the minority class or by reducing the number of cases in the majority class [16].

Over-sampling techniques applied to the minority class present several notable challenges within the medical domain. Basic approaches, such as random duplication of existing cases, may lead to model overfitting [17], where the model overly relies on a limited subset of variables. Modern generative methods, such as SMOTE [17] or similar algorithms that dynamically synthesize new data based on the patterns of the given cases, may further introduce ethical and legal concerns. Recent evidence shows that SMOTE is inherently non-private [18]: adversaries can mount effective membership inference attacks and even reconstruct real minority-class records from SMOTE-generated datasets, meaning the very individuals represented by rare conditions or under-represented subpopulations may become disproportionately exposed.

Beyond privacy, synthetic augmentation may amplify existing structural biases and distort clinically meaningful relationships, particularly for intersectional subgroups, yielding spurious or missing associations and increasing “synthetic trust” in models trained on artificially generated data that do not preserve demographic or clinical realities [19,20].

Given the limitations associated with over-sampling methodologies, this study applied random under-sampling to achieve class balance by selectively eliminating some non-URV cases. In contrast to over-sampling approaches, random under-sampling offers a more reliable strategy by reducing the number of non-URV records without introducing synthetic data, therefore preserving the integrity of the dataset. This approach facilitates a more proportionate class distribution, enhances the performance of the predictive model, and mitigates the risk of bias arising from class imbalance.

### 3.5. Prediction Model

Convolutional Neural Network (CNN) is a classification model that can fully leverage both structured and unstructured data. Its architecture, composed of convolutional, pooling, and fully connected layers, is particularly effective in automatically extracting hierarchical representations from complex data sources. In the medical domain, CNNs are capable of capturing latent patterns within unstructured data such as clinical notes, while integrating structured variables including vital signs, laboratory results, and demographic information at the same time. Such ability makes CNN a suitable framework for predictive tasks such as ED readmission, where clinically relevant features may be distributed across multiple data modalities.

This study employed two separate CNN models: one tailored for structured data and the other for unstructured data. This multimodal approach allows each subnetwork to specialize in feature extraction tailored to its specific data modality before the final decision-making process. The branch dedicated to structured variables utilizes a 1D-CNN architecture characterized by 64 filters and a kernel size of 3. This stream incorporates ten 1D Max-Pooling layers with a pooling size of 2, enabling the model to distill high-level patterns from clinical tabular data. In contrast, the unstructured variables branch employs a hierarchical configuration with filter sizes of 256, 128, and 64, respectively. A larger kernel size of 5 and three 1D Max-Pooling layers were utilized here to capture complex semantic dependencies and linguistic nuances within medical notes. Both streams employ the Rectified Linear Unit (ReLU) as the primary activation function to introduce non-linearity while maintaining computational efficiency.

Both models are subsequently integrated through a concatenation layer. To ensure stable and robust convergence, the model was trained using the NAdam optimizer with a batch size of 256. This study implemented a dynamic learning rate strategy via the ReduceLROnPlateau scheduler. Additionally, this study employed an early-stopping mechanism with a patience value of 25 to minimize the possibility of model overfitting. Training is automatically terminated if no further improvement in performance is observed after 25 consecutive epochs, thereby preventing excessive weight adaptation to specific variables and reducing unnecessary computational cost.

Table 4 illustrates the architecture of the proposed CNN-based prediction model, and Table 5 summarizes the optimized training hyperparameters.

### 3.6. Model Interpretation

DL-based models are often regarded as black boxes, as the inherent structural complexity obscures the detailed decision-making process. The lack of interpretability poses a major challenge to clinical adoption, as clinicians require transparent reasoning to trust and validate model outputs. To address this limitation, this study applied Shapley values (SHAP) to enhance model interpretability. SHAP quantifies the marginal contribution of each variable by assessing its impact across different feature subsets, thereby providing a consistent and theoretically grounded explanation of model predictions. In the medical context, such interpretability not only enables clinicians to understand which variables most strongly influence predictive outcomes but also facilitates the identification of potential biases, improves trust in automated decision support, and supports the alignment of predictive results with established clinical knowledge.

## 4. Results

This study conducted a series of experiments to evaluate the effectiveness and efficiency of the proposed model:

1. Experiment 1: Assessed the impact of transforming continuous numeric variables into binary representations.
2. Experiment 2: Benchmarked the proposed framework against a range of algorithms based on both ML and DL.
3. Experiment 3: Demonstrated model interpretability using SHAP analysis.

### 4.1. Dataset and Ethical Considerations

Kaohsiung Veterans General Hospital (KVGH) is one of the largest hospitals in Taiwan, providing inpatient, outpatient, and ED services to the southern region. This study analyzed retrospective administrative medical records of outpatients who sought ED care at KVGH. The participant selection process began with an initial pool of 184,687 ED outpatients treated between 1 January 2018, and 31 December 2022. To ensure data integrity, a primary screening criterion was implemented requiring at least one documented valid ED admission. Following the exclusion of 34 patients who failed to meet this requirement, the final study cohort comprised 184,653 patients. This analytical population was further stratified into 175,816 non-URV cases and 8837 URV cases for model training and evaluation.

The study was conducted in accordance with the principles of the Declaration of Helsinki and was approved by the Institutional Review Board of Kaohsiung Veterans General Hospital (IRB certification number: KSVGH23-CT5-04). All data were fully de-identified, ensuring that no individual could be identified. The rights and interests of the patients were safeguarded, and the study had no impact on their treatment or medication, either prior to or following data analysis.

### 4.2. Performance Matrix

This study employed three performance indicators to evaluate the effectiveness of the proposed models: accuracy, sensitivity (also known as recall), and the area under the Receiver Operating Characteristic curve (AUROC). Accuracy reflects the overall proportion of correctly classified cases. Recall measures the ability of the model to identify positive cases, which is particularly important in medical applications where missing true cases may lead to adverse outcomes. AUROC assesses the model’s discriminative capacity across different classification thresholds, with higher values indicating superior overall performance in distinguishing between positive and negative cases.

To develop and evaluate the prediction models, the dataset was randomly divided into training and testing subsets using an 8:2 split. To account for variability arising from random data partitioning, 10-fold Monte Carlo Cross Validation was employed in experimental runs, each employing a distinct random split of the data. Model performance was then assessed by averaging the evaluation metrics obtained from these ten runs, thereby providing a more robust and reliable estimate of predictive performance while reducing the influence of sampling variance.

To rigorously account for the class imbalance inherent in predicting URV, the 95% confidence intervals (CIs) for both accuracy and sensitivity were estimated using the Clopper–Pearson Exact method. Given the low prevalence of the minority class in our dataset, traditional asymptotic approximations, such as the Wald interval, often lack reliability as proportions approach the boundaries of zero or one. By employing the Clopper–Pearson approach, which is derived directly from the cumulative binomial distribution rather than a normal approximation, it provides a more conservative and statistically robust assessment of model performance, reinforcing the clinical credibility of the predictive outcomes.

### 4.3. Experiment 1: Effect of Binary Transformation of Continuous Variables

Data representation plays a critical role in determining how predictive models interpret input features, and different representation strategies can result in substantial variations in model performance. In medical applications, even minor numerical differences in physiological measurements may correspond to markedly different clinical interpretations. For instance, body temperatures of 35 °C and 36 °C are generally considered within the normal range, whereas a temperature of 37 °C is often regarded as indicative of fever in clinical practice. Such threshold-based distinctions motivate the exploration of alternative feature representations that better align with clinical reasoning.

This experiment investigated the impact of transforming continuous numerical variables into binary representations on predictive performance. The primary objective is to assess whether binary transformation can preserve clinically meaningful distinctions while enhancing the robustness of the predictive model. The experimental results are summarized in Table 6.

The findings indicate that the binary transformation led to improved predictive robustness, as evidenced by increases in both Recall and the AUROC. Specifically, sensitivity increased from 0.709 to 0.717, and AUROC improved marginally from 0.852 to 0.853, suggesting enhanced sensitivity and discriminative capability of the model. Although a slight decrease in overall accuracy was observed (from 0.860 to 0.846), this trade-off may be acceptable in clinical settings where sensitivity is often prioritized over raw accuracy. Additionally, the binary representation incurs a longer training time, increasing from 263 s to 339 s, reflecting the computational cost associated with the transformation. Overall, the experimental results demonstrate that binary transformation is capable of preserving clinically relevant distinctions while improving key robustness-related performance metrics with minor and acceptable trade-offs in accuracy and training time.

### 4.4. Experiment 2: Comparative Evaluation of ML- and DL-Based Algorithm

This section evaluates the predictive performance of different algorithms to determine the relative effectiveness of alternative modeling approaches for URV prediction [11,21]. All the comparison models were trained using the same multimodal information (structured and unstructured data) and tuned to the optimal performance.

For ML-based models, Support Vector Machine (SVM), Lasso Regression (LR), and Random Forest (RF) were selected, as they are widely adopted in medical informatics research and have demonstrated robustness in handling high-dimensional and heterogeneous clinical data.

For DL-based models, CNN, LSTM, and MLP were employed. These architectures are commonly applied in the medical domain, with CNN particularly effective in extracting complex patterns from unstructured information, while MLP has shown strong performance in learning from structured variables. The comparative results of these experiments are summarized in Table 7.

The results demonstrate that DL-based algorithms consistently outperformed ML-based models across all evaluation metrics. The superior predictive performance of DL techniques may be attributed to their more complex architectures, which enable the extraction of latent relationships among variables and the modeling of non-linear patterns. It is noteworthy, however, that RF required substantially less training time while still achieving reasonably acceptable predictive performance, whereas SVM showed no advantage in computational efficiency and performed inferiorly compared to the other models.

Within the DL-based models, MLP achieved higher accuracy compared to CNN, indicating a stronger overall correctness in classification. However, CNN outperformed MLP in both sensitivity and AUROC, suggesting superior capability in identifying URV cases and in overall discriminative performance across different thresholds. A possible explanation lies in the architectural characteristics of CNN, which allow for a more effective extraction of complex and latent feature representations, particularly from unstructured clinical data. In contrast, MLP relies primarily on structured variable learning, which may limit its ability to capture the non-linear dependencies and heterogeneous patterns present in multimodal medical datasets.

### 4.5. Experiment 3: Model Interpretability Analysis Using SHAP

**Figure 2:**
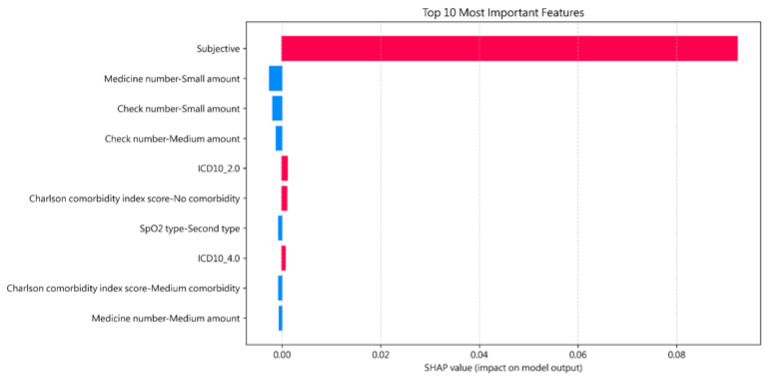
SHAP analysis results. Red denotes positive impacts on the prediction; blue denotes negative impacts.

This experiment demonstrates the interpretability of the proposed model through the application of SHAP, with the objective of identifying key predictive variables and enhancing both transparency and clinical applicability. SHAP was employed to conduct a global feature importance analysis across the entire dataset, therefore revealing the overall decision logic of the model and the primary variables that underpinned its predictions. Figure 2 is the SHAP analysis results.

In this visualization, features represented by red bars indicate a positive impact on the model’s decision-making process, indicating that these variables increase the predicted probability of URV. Conversely, blue bars denote features with a negative impact, suggesting that their presence or specific values serve as inhibitory factors that lower the likelihood of URV in the model’s output.

The SHAP analysis results reveal that the feature “Subjective”, which is embedded and derived from unstructured medical notes, exerts a dominant influence on the model’s predictive outcomes, far exceeding the contribution of any other variable. Physicians observed that the high importance of the ‘Subjective’ feature is clinically sound; patients with severe conditions or complex family histories tend to have more extensive documentation in their medical notes, which the model correctly identified as a risk factor. This finding highlights the significant role of unstructured clinical text in the decision-making process of the model. The importance of this feature further validates that unstructured data contain rich semantic information, which substantially enhances the model’s discriminative capacity.

Additionally, the model’s identification of “small amounts” of medications or laboratory checks as negative predictors for URV was viewed as highly intuitive. Clinicians confirmed that patients requiring minimal pharmacological intervention or fewer diagnostic tests typically represent lower-acuity cases with a naturally reduced risk of return visits.

### 4.6. Small-Scale Clinical Pilot Evaluation and Qualitative Feedback

**Figure 3:**
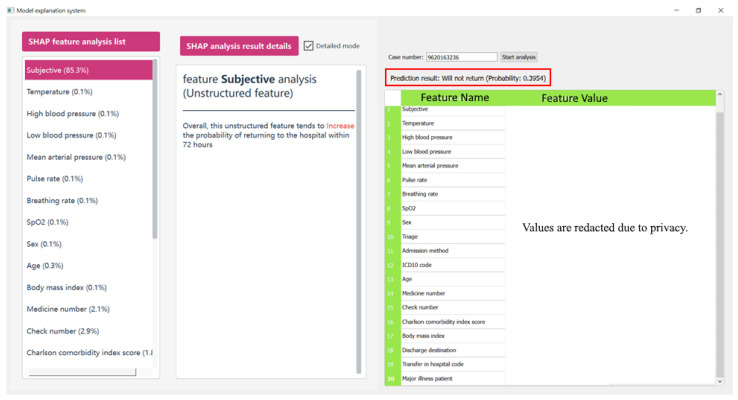
Bipartite user interface.

To translate the predictive model into a practical clinical tool, this study designed a bipartite user interface that prioritizes both transparency and clinical context. As shown in Figure 3, the interface is divided into two primary functional modules:

- SHAP Interpretability Dashboard (Left Panel): This module provides a breakdown of the model’s decision logic through the “SHAP feature analysis list”. It ranks clinical variables based on their contribution to the specific prediction, such as the prominent influence of the ‘Subjective’ notes (85.3%). By clicking on individual features, clinicians can access detailed mode-specific explanations, helping them understand why the system flagged a patient as high-risk.
- Patient Clinical Profile (Right Panel): To support rapid verification, the right side of the interface displays comprehensive patient data, including demographics, vital signs, and medication history. This allows physicians to immediately cross-reference the model’s risk assessment with the raw clinical evidence.

To validate the practical utility of the proposed framework within a real-world clinical setting, a small-scale pilot evaluation was conducted at our collaborating medical institution. This study utilized a survey-based methodology to gather qualitative insights from participating physicians, structured around two core investigative questions:

1. Clinical Utility: Does the system provide actionable assistance in the identification and prediction of unplanned return visits?
2. Trustworthiness: To what degree are the data and predictive logic provided by the system considered credible for clinical decision-making?

Regarding the first dimension, clinicians noted that while the model occasionally yielded false positives or negatives, it exceled at identifying high-risk patient cohorts that might otherwise be overlooked. A key highlight was the system’s ability to aggregate and present relevant patient data for immediate comparison. Physicians reported that this consolidated view streamlined the clinical assessment process, effectively reducing the cognitive workload previously required to cross-reference multiple disparate hospital information systems. Regarding the second dimension, a critical barrier to the adoption of previous predictive tools has been their “black box” nature. Our framework addresses this by incorporating SHAP-based interpretability.

By bridging the gap between prediction and clinical reasoning, these SHAP-derived insights confirmed that the decision-making process of proposed framework is both transparent and medically plausible, therefore enhancing greater confidence in its clinical application.

## 5. Discussion

This study proposes a short-term solution to ED overcrowding by developing a DL-assisted CDSS for predicting patients at risk of ED revisits by leveraging features from both structured and unstructured medical data. This study further presents figures illustrating model interpretation through SHAP. By quantifying the contribution of each variable to the model’s output, the global SHAP analysis identifies the variables exerting the greatest influence on predictive performance. These insights enhance the interpretability and credibility of the model and therefore build clinician trust and support the potential integration of the model into real-world medical decision-making.

For practical implementation, AUROC was adopted as the primary indicator of overall model performance. Models achieving higher AUROC values are associated with fewer false alerts while maintaining an acceptable true positive rate. In scenarios where the complete identification of URV cases is prioritized, recall serves as a critical supplementary metric. The experimental results indicate that the CNN model outperforms alternative approaches in terms of both recall and AUROC, whereas RF may serve as a reasonably effective and computationally efficient alternative when resources or training time are limited.

The proposed framework effectively predicts the probability of URV while providing reasonable attributions for model decisions. By reducing the likelihood of URV, the framework may contribute to mitigating ED overcrowding and enhancing the sustainability of ED operations, serving as a practical short-term strategy prior to the implementation of long-term systemic solutions.

## 6. Research Limitations and Future Work

This study was conducted using retrospective emergency department EHR data collected between 2018 and 2022 from a single medical center. As a result, the data may reflect institution-specific case mix, documentation practices, and operational workflows. Our primary contribution is a modeling and deployment framework. Because ED processes and EHR representations vary across institutions, model parameters trained at one site may not directly transfer to another without local adaptation. Methodologically, model development used an 8:2 random split and 10-fold Monte Carlo cross-validation, which supports internal performance estimation under random resampling. However, the evaluation was not explicitly temporally separated, and potential temporal distribution shift was not directly assessed. This study provides uncertainty using Clopper–Pearson exact confidence intervals, which quantify variability within the study sample but do not, on their own, establish transportability across institutions or time periods.

To resolve these limitations, future work will focus on translating the proposed framework into real-world application, including practical integration into clinical workflows and ongoing performance monitoring in routine use.

## 7. Conclusions

ED overcrowding represents a critical challenge to healthcare sustainability, severely compromising both clinical care quality and operational efficiency. While systematic resolution of this issue necessitates long-term policy reforms and administrative restructuring, this study provides a more immediate, practical intervention to mitigate these pressures. By developing a Deep Learning-assisted CDSS that integrates both structured and unstructured medical data, this study offers a robust framework for identifying patients at high risk of ED revisit.

The evaluation findings yield several key insights for clinical implementation. First, this study demonstrates that data representation is fundamental to predictive performance; specifically, the binary transformation of continuous numeric data enhances model robustness, as reflected in superior Recall and AUROC metrics. Second, while the CNN model proved to be the most effective architecture for this task, the Random Forest model remains a viable, computationally efficient alternative for resource-constrained environments. Finally, the application of SHAP analysis indicates the indispensable value of unstructured clinical text in refining the model’s decision-making process. Collectively, these results provide a scalable short-term solution to alleviate ED congestion and support clinicians in resource-heavy environments.

## Figures and Tables

**Table 1:** The demographic of the structured variables in the studied dataset.

Variables	Data Range
Age	17–111 (year old)
Mean Arterial Pressure	17–230 (mmHg)
Systolic Blood Pressure	12–279 (mmHg)
Diastolic Blood Pressure	6–227 (mmHg)
Pulse Rate	5–275 (BPM)
Body Temperature	25–44 (°C)
Oxygen Saturation	51–100 (%)
Respiration Rate	1–79 (breathe per minute)
Number of Laboratory Check Results	0–75
Number of Medicine Categories	0–26

**Table 2:** Reference Interval.

Vital Signs	Reference Interval
Mean Arterial Pressure	70~100 (mmHg)
Systolic Blood Pressure	90~120 (mmHg)
Diastolic Blood Pressure	60~80 (mmHg)
Pulse Rate	60~100 (bpm)
Body Temperature	35~38 (°C)
Respiration Rate	12~20 (bpm)
Oxygen Saturation	95~100 (%)

**Table 3:** The demonstration of one-hot encoding representations.

**Before OHE**		→	**After OHE**		
**Patient**	**Disease**		**Diabetes**	**Rhinitis**	**Epilepsy**
A	Diabetes		1	0	0
B	Rhinitis		0	1	0
Γ	Rhinitis		0	1	0
Δ	Epilepsy		0	0	1

**Table 4:** The CNN architecture in the proposed CDSS.

Architecture of the Proposed CNN Model	
CNN model architecture for structured variables	
1D Convolutional Neural Network	
1D Max Pooling × 10	
Filter value	64
Kernel size	3
Pooling size	2
Activation Function	Rectified Linear Unit
CNN model architecture for unstructured variables	
1D Convolutional Neural Network	
1D Max Pooling × 3	
Filter value	256, 128, and 64, respectively
Kernel size	5
Pooling size	3
Activation Function	Rectified Linear Unit
Concatenate Layer	
Dense (Fully Connected) Layer	

**Table 5:** The optimized training hyperparameters.

Hyperparameter	Value
Batch size	256
Epoch	100 (with early stopping)
Patience	25
Learning rate scheduler	ReduceLROnPlateau
Optimizer	NAdam
Learning rate scheduler parameters	
Factor	0.5
Patience	5
Minimal learning rate	1 × 10^−4^
Optimizer parameters	
Learning rate	Dynamic
Epsilon	1 × 10^−4^

**Table 6:** The results between original representation and binary transformation.

	Original Representation	Binary Transformation
Accuracy	0.860 [0.856, 0.864]	0.846 [0.842, 0.850]
Sensitivity	0.709 [0.687, 0.730]	0.717 [0.695, 0.738]
AUROC	0.852	0.853
Train Time	263.160 s	339.251 s

**Table 7:** The comparative results of various models.

Model	Accuracy	Sensitivity	AUROC	Time (s)
CNN	0.846 [0.843, 0.850]	0.717 [0.695, 0.738]	0.853	339.251
MLP	0.856 [0.852, 0.860]	0.633 [0.610, 0.656]	0.822	689.137
LSTM	0.864 [0.860, 0.867]	0.540 [0.516, 0.563]	0.651	413.541
RF	0.656 [0.651, 0.661]	0.668 [0.633, 0.678]	0.723	68.431
SVM	0.630 [0.625, 0.635]	0.477 [0.454, 0.500]	0.572	311.596
LR	0.731 [0.726, 0.736]	0.448 [0.425, 0.472]	0.610	60.861

## Data Availability

Datasets are not publicly available due to privacy and ethical constraints; access may be considered upon application to Kaohsiung Veterans General Hospital.

## Abbreviations

The following abbreviations are used in this manuscript:

AUC	Area Under the Curve
AUROC	Area Under the Receiver Operating Characteristic Curve
BMI	Body Mass Index
CCI	Charlson Comorbidity Index
CDSS	Clinical Decision Support System
CNN	Convolutional Neural Network
DL	Deep Learning
ED	Emergency Department
EHR	Electronic Health Record
ICD-10	The International Statistical Classification of Diseases and Related Health Problems 10th Revision
MAP	Mean Arterial Pressure
ML	Machine Learning
MLP	Multilayer Perception
NHI	National Health Insurance
OHE	One-Hot Encoding
RF	Random Forest
SHAP	Shapley Value
SVM	Support Vector Machine
URV	Unexpected/Unscheduled Return Visit
